# Ubiquitin D Promotes Lung Metastasis by Stabilizing MMP3 in Triple-Negative Breast Cancer

**DOI:** 10.34133/research.1065

**Published:** 2026-01-23

**Authors:** Rong Xu, Tao Wu, Hailong Li, Shixiang Ji, Qi Zhou, Yun Peng, QiangQiang Zhao, Xiaoqing Sun, Peng Liu, Wei Du

**Affiliations:** ^1^ Department of Pathology, Changde Hospital, Xiangya School of Medicine, Central South University (The First People’s Hospital of Changde City), Changde, Hunan 415000, China.; ^2^ State Key Laboratory of Oncology in South China, Guangdong Provincial Clinical Research Center for Cancer, Sun Yat-sen University Cancer Center, Guangzhou, Guangdong 510060, China.; ^3^ Department of Oncology, Changde Hospital, Xiangya School of Medicine, Central South University (The First People’s Hospital of Changde City), Changde, Hunan 415000, China.; ^4^ Department of Hematology, Liuzhou People’s Hospital affiliated to Guangxi Medical University, Liuzhou, Guangxi 545000, China.; ^5^ Department of Hematology, The Qinghai Provincial People’s Hospital, Xining, Qinghai 810000, China.

## Abstract

Triple-negative breast cancer (TNBC) represents a notably aggressive form of breast cancer, distinguished by heightened invasiveness and an important propensity for metastasis. The expression of ubiquitin D (UBD) is significantly increased in lung metastases associated with TNBC, correlating with unfavorable patient outcomes. Functional assays indicate that UBD promotes invasion, migration, and pulmonary colonization of TNBC cells in vivo. At the mechanistic level, UBD preserves matrix metalloproteinase 3 (MMP3) levels by inhibiting proteasomal degradation. A proteomic analysis has identified MMP3 as a crucial downstream mediator of UBD. Concurrently, chromatin immunoprecipitation and luciferase reporter assays demonstrate that the Spi-B transcription factor (SPIB) directly interacts with the UBD promoter, leading to the activation of its transcription. Collectively, these findings identify the SPIB/UBD/MMP3 axis as a pivotal regulator of TNBC metastasis, indicating its value for prognostic evaluation and targeted therapy.

## Introduction

Triple-negative breast cancer (TNBC), characterized by lacking all 3 receptors, represents the most aggressive breast cancer subtype [1–4]. With a high degree of invasiveness, TNBC tends to metastasize to far-off organs like the liver, brain, lungs, and bones [5–7]. While bone represents the most common site for metastasis across all breast cancer subtypes, TNBC demonstrates a heightened inclination toward lung metastasis, affecting approximately 32% of TNBC patients, in contrast to around 21% in luminal A/B and 25% in human epidermal growth factor receptor 2 (HER2)-positive cases [8]. However, the specific mechanisms through which TNBC facilitates lung metastasis remain inadequately understood.

Posttranslational protein modification through ubiquitin and ubiquitin-like modifiers (ULMs) is vital for managing protein stability, cellular localization, functionality, and protein–protein interactions [9]. Among these ULMs, ubiquitin D (UBD) or FAT10, is a ULM that resides in the major histocompatibility complex (MHC) class I region and activated by cytokines like tumor necrosis factor-α (TNF-α) and interferon-γ (IFN-γ) [10,11]. Unlike ubiquitin, which requires a sequential cascade of E1, E2, and E3 enzymes for substrate conjugation, FAT10 is processed through a distinct and less canonical system. FAT10ylation is mediated by its dedicated E1 enzyme UBA6 and the E2 enzyme USE1/UBE2Z, but a universally defined E3 ligase has not been identified [12]. Emerging evidence suggests that specific E3s may contribute in a substrate- or context-dependent manner—for example, Parkin has been reported to promote FAT10ylation of selected substrates [13], and TRIM25 has been implicated under certain conditions [14]. Thus, in contrast to ubiquitination, FAT10 conjugation lacks a general E3 ligase, and its regulation appears to be highly context-specific.

Although UBD was initially investigated in the realm of immune regulation, accumulating evidence links its aberrant expression to tumorigenesis and cancer progression [15–17]. Recent pan-cancer analyses have further underscored the broad relevance of UBD in malignancy, including glioma, colorectal, hepatocellular, and breast cancers, and has been shown to correlate with prognosis, immune infiltration, and various clinical characteristics [18]. Notably, UBD exhibits dual molecular functions within tumor cells—serving both degradative and stabilizing roles [19,20]. This apparent duality reflects the context-dependent nature of UBD conjugation. In some settings, UBD acts as a degradation signal by recruiting substrates to the proteasome through interactions with NUB1 or NUB1L, leading to their ubiquitin-independent degradation [21,22]. In other contexts, FAT10 conjugation can competitively block ubiquitination or stabilize proteins by masking lysine residues, thereby preventing their proteasomal turnover [23].

For instance, UBD shows elevated expression levels in breast cancer, and its down-regulation has been found to inhibit epithelial–mesenchymal transition (EMT) and the metastatic behavior of breast cancer cells, possibly through the stabilization of the ZEB2 protein [24]. This indicates that UBD may play a pivotal oncogenic role in the advancement of breast cancer and could represent a viable prognostic and therapeutic target. In contrast, in hepatocellular carcinoma, it contributes to tumorigenesis via the degradation of p53 [16]. Nonetheless, the molecular mechanisms responsible for UBD up-regulation in breast cancer are not well understood. Specifically, the functional role and regulatory pathways of UBD in fostering distant metastasis in TNBC, particularly to the lungs, have yet to be comprehensively explored.

Matrix metalloproteinases (MMPs) are zinc-dependent proteolytic enzymes essential for the remodeling of the extracellular matrix (ECM) and have been extensively implicated in cancer cell invasion and metastatic progression [25]. Highly metastatic TNBC cells have the capability to transfer MMP-1 through exosomes, which activates PAR1-mediated EMT, thereby enhancing the distant metastasis of cells with low metastatic potential [26]. Among these, MMP3 has been demonstrated to facilitate EMT, increase cell motility, and promote metastatic spread in a variety of cancers [27,28]. Although the transcriptional regulation of MMP3 has been thoroughly investigated, the posttranslational mechanisms that stabilize MMP3 in TNBC remain to be clarified.

In this investigation, we provide evidence that UBD expression is markedly up-regulated in TNBC and is linked to adverse clinical outcomes and the incidence of lung metastases. From a mechanistic perspective, Spi-B transcription factor (SPIB) is a crucial transcription factor responsible for the up-regulation of UBD. Additionally, our research demonstrates that UBD has the capacity to interact with and stabilize MMP3, which in turn facilitates EMT and promotes the metastatic potential of TNBC cells to the pulmonary system. Collectively, the results reveal a novel SPIB/UBD/MMP3 signaling pathway that is pivotal in TNBC progression, proposing UBD as a promising candidate for therapeutic intervention in metastatic TNBC.

## Results

### UBD is up-regulated in TNBC and serves as an unfavorable prognostic factor

**Fig. 1.**
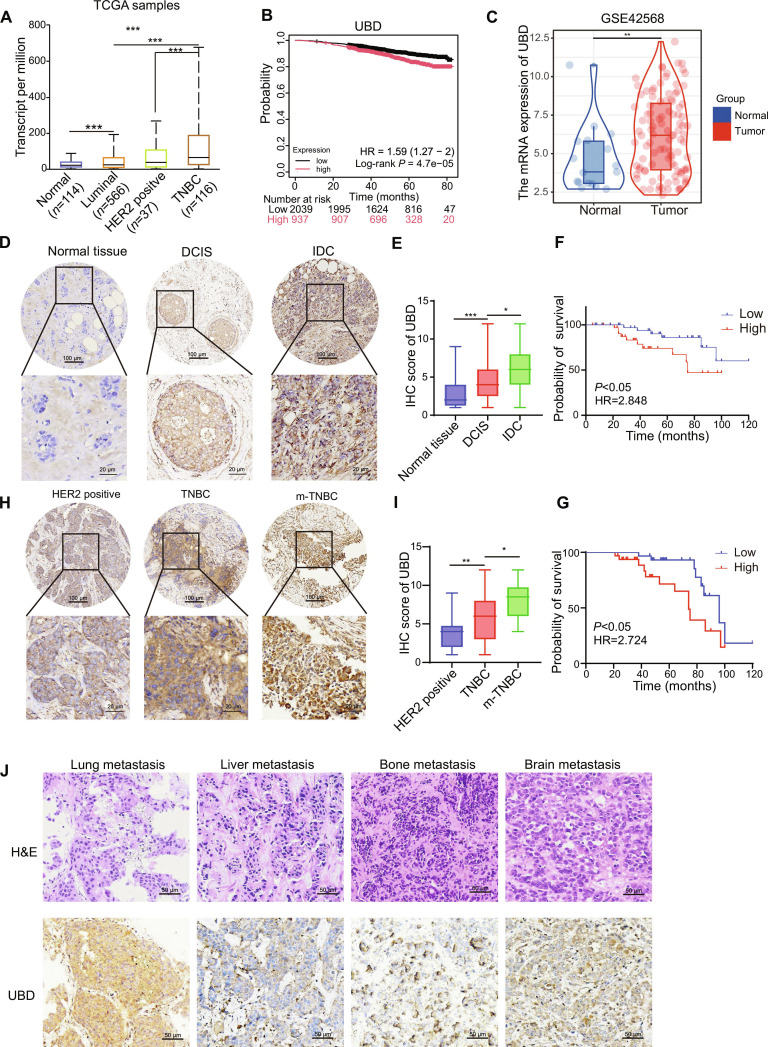
UBD is up-regulated in TNBC and as an unfavorable factor for TNBC. (A) The UALCAN database was utilized to examine the expression levels of UBD across various subtypes of breast cancer. (B) The relationship between UBD and survival among breast cancer patients was analyzed via the Kaplan–Meier Plotter database. (C) GSE42568 was used to analyze the expression of UBD. (D and E) Expression patterns in breast cancer tissues with different histological subtypes. (F and G) Overall survival (OS) and disease-free survival (DFS) rates were evaluated and contrasted between groups exhibiting high levels of UBD expression and those with low levels of UBD expression. ***P < 0.001. (H and I) Expression patterns in TNBC and metastatic TNBC (m-TNBC), including primary and metastatic TNBC. (J) UBD expression in metastatic TNBC tissues.

To evaluate the expression profiles and clinical relevance of ULMs in TNBC, we initially assessed their expression profiles utilizing the UALCAN and Kaplan–Meier Plotter databases. The ULMs selected for this analysis comprised SUMO1, SUMO2, SUMO3, ISG15, NEDD8, UFM1, URM1, ATG12, and UBD. The high levels of SUMO2, ISG15, NEDD8, and UBD are related to worse prognosis outcomes in breast cancer (Fig. S1 and Fig. 1A and B). Furthermore, SUMO2 and UBD are up-regulated in TNBC tissues than in luminal and HER2-positive subtypes. To corroborate these results, we conducted an examination of the GSE42568 dataset, which confirmed that UBD levels were significantly higher in breast cancer tissues (Fig. 1C). Subsequently, immunohistochemical (IHC) analyses were performed on 80 breast cancer specimens alongside adjacent normal tissues, demonstrating that UBD was expressed at significantly higher levels in invasive tumor tissues as opposed to adjacent normal tissues and ductal carcinoma in situ (Fig. 1D and E). IHC observations in both ductal carcinoma in situ and invasive carcinoma are illustrated in Fig. S2A. Notably, elevated UBD expression was linked to poorer survival outcomes for breast cancer patients (Fig. 1F and G). Furthermore, expression of UBD was substantially elevated in breast cancer tissues when compared to normal tissues, which aligns with the IHC findings presented in Fig. S2B.

Moreover, UBD protein levels were significantly heightened in metastatic TNBC tissues in comparison to nonmetastatic TNBC (Fig. 1H and I). An evaluation of UBD expression across different metastatic sites revealed notably increased levels in lung metastases of breast cancer as opposed to other distant metastases (Fig. 1J). A summary of the relationship between UBD expression and clinicopathological factors is provided in Table S1. Elevated UBD levels were significantly associated with advanced tumor–node–metastasis (TNM) staging (*P* = 0.023), increased Ki67 expression (*P* = 0.017), and distant metastasis (*P* = 0.038). Finally, we further analyzed UBD expression in TNBC metastatic samples to various organs, including the liver (*n* = 30), lung (*n* = 10), brain (*n* = 6), and bone (*n* = 6). Additionally, in the Cox regression analysis, we found that the *P* value for high UBD expression was less than 0.05, indicating that UBD expression significantly impacts patient survival and can be considered an independent prognostic factor (Table S3).

### UBD promotes cell proliferation and migration in TNBC

**Fig. 2.**
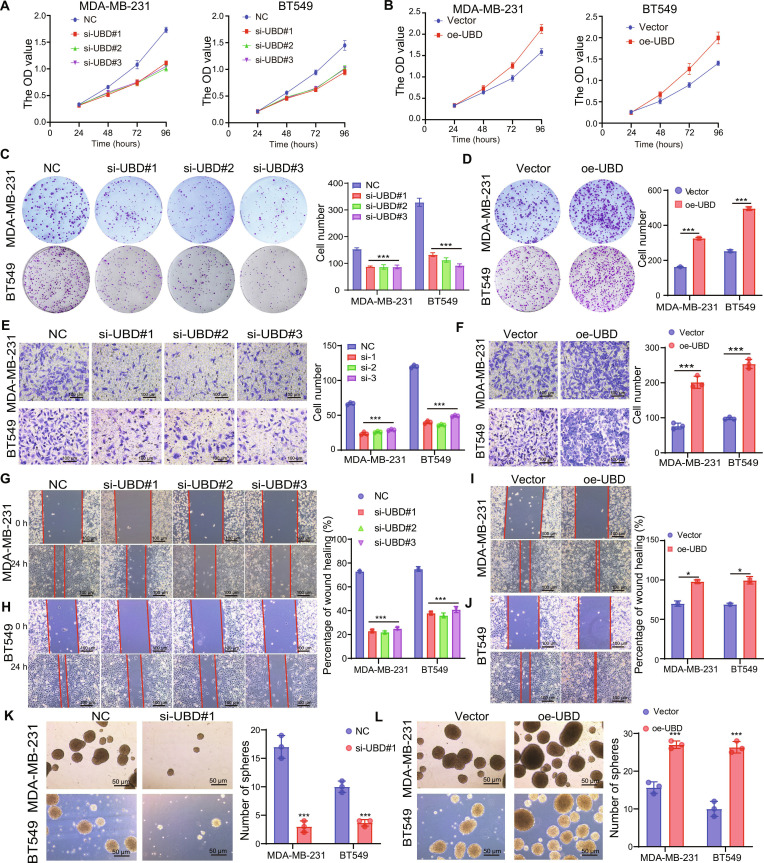
UBD promotes the migration and maintain cancer stemness in TNBC. (A to D) CCK8 assay and colony formation assessed cell proliferation. (E and F) Transwell assay analysis cell migration ability. (G to J) Wound healing assay further determined the effect of UBD on migratory potential. (K and L) Tumor sphere formation was used to analyze the effect of UBD on maintaining tumor stemness. ***P < 0.001.

To investigate the potential role of UBD in TNBC, quantitative polymerase chain reaction (qPCR) was performed to assess small interfering RNA (siRNA) transfection efficiency, as shown in Fig. S3A and B. Cell Counting Kit-8 (CCK8) analysis indicated that UBD knockdown significantly suppressed cell proliferation in cells, whereas UBD overexpression promoted cell proliferation (Fig. 2A and B).

Furthermore, the colony formation assay confirmed that UBD knockdown inhibited colony formation ability, while its overexpression enhanced it (Fig. 2C and D). As shown in Fig. 2E and F, UBD knockdown significantly reduced migration ability, whereas UBD overexpression enhanced it. Similarly, wound healing assays revealed that UBD knockdown suppressed TNBC cell migration, whereas UBD overexpression had the opposite effect (Fig. 2G to J).

Cancer stem cells (CSCs) are identified based on their self-renewal potential and their potential for limitless proliferation, attributes that significantly contribute to the aggressive nature of tumors and the unfavorable clinical outcomes observed in patients [29,30]. A 3-dimensional (3D) tumor sphere formation assay was performed to evaluate the effect of UBD on clonogenicity. Tumor sphere size and number were significantly reduced in the si-UBD group (Fig. 2K to M).

Analysis using the CancerSEA database indicated that UBD is associated with stemness and positively correlated with stemness and EMT-related traits (Fig. S4A and B). We selected N-cadherin and E-cadherin as classical EMT markers, since the down-regulation of E-cadherin and the up-regulation of N-cadherin reflect the EMT, a critical step driving tumor invasion and metastasis. Similarly, CD44 and CD133 were chosen as representative CSC markers, as their expression denotes self-renewal capacity and tumor-initiating potential. Additionally, the knockout of UBD resulted in a reduction of levels of CD44 and CD133, alongside a decrease in N-cadherin. Conversely, there was an elevation in the expression of E-cadherin (Fig. S4C and D).

### Knockdown of UBD suppresses in vivo tumor growth and lung metastasis in xenograft models

**Fig. 3.**
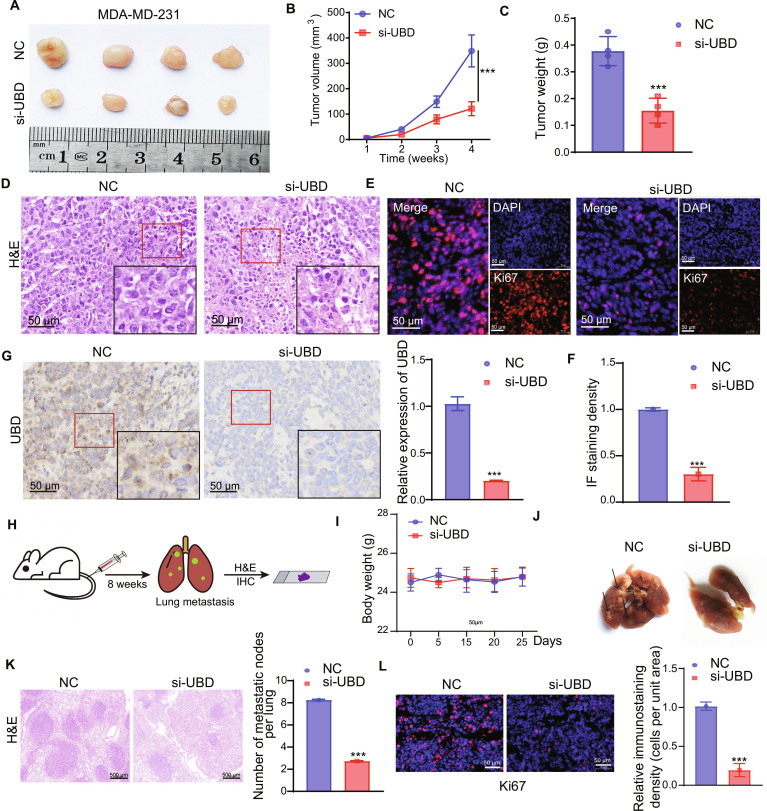
Knockdown UBD suppresses tumor progression and in vivo lung metastasis of TNBC. (A) Representative images from the control group (top) and experimental group (bottom) show differences in tumor size and morphology (*n* = 4). (B) Tumor volumes were quantified by *V* = *L* × *W*^2^/2. (C) Tumor weight analysis in NC and si-UBD groups. (D) H&E staining of representative tumor in NC and si-UBD groups. (E and F) Representative images of immunofluorescence staining for Ki67 in NC and si-UBD groups. (G) Immunofluorescence staining for Ki67 in NC and si-UBD groups. (H) Schematic of the xenograft-induced metastasis model, with lung metastases assessed by H&E and IHC. (I) Body weight of mice in NC and si-UBD groups. (J) Metastatic sites in the lung were noted to be decreased in the si-UBD groups (*n* = 4). (K) H&E assay used to display the metastatic nodes. (L) Representative immunostaining images for Ki67 of lung metastasis resulting from NC and si-UBD. Results are reported as mean ± SD. ***P < 0.001.

A subcutaneous tumor experiment was carried to further explore the function of UBD in vivo. As shown in Fig. 3A, UBD in vivo siRNA significantly reduced tumor growth. Additionally, tumor volume, in terms of both volume and weight, was markedly decreased following UBD in vivo siRNA treatment (Fig. 3B and C). Hematoxylin and eosin (H&E) staining indicated that UBD knockdown led to a reduction in tumor cell population while simultaneously enhancing apoptotic activity (Fig. 3D). Immunofluorescence and IHC assays further demonstrated that Ki67 and UBD expression levels were decreased in tumor tissues treated with UBD in vivo siRNA (Fig. 3E and F).

To evaluate the function of UBD in TNBC metastasis, a murine lung metastasis model was generated by injecting MDA-MB-231 cells transfected with either UBD siRNA or control siRNA (Fig. 3H). The groups exhibited no notable differences in terms of body weight (Fig. 3I). However, UBD knockdown significantly reduced the number of pulmonary metastatic nodules (Fig. 3J and K). Furthermore, Ki67 expression in lung tissues was assessed, revealing that UBD knockdown decreased Ki67 levels in the TNBC lung metastasis model treated with UBD in vivo siRNA compared to the control siRNA group (Fig. 3L).

### UBD stabilizes MMP3 protein in TNBC cells

**Fig. 4.**
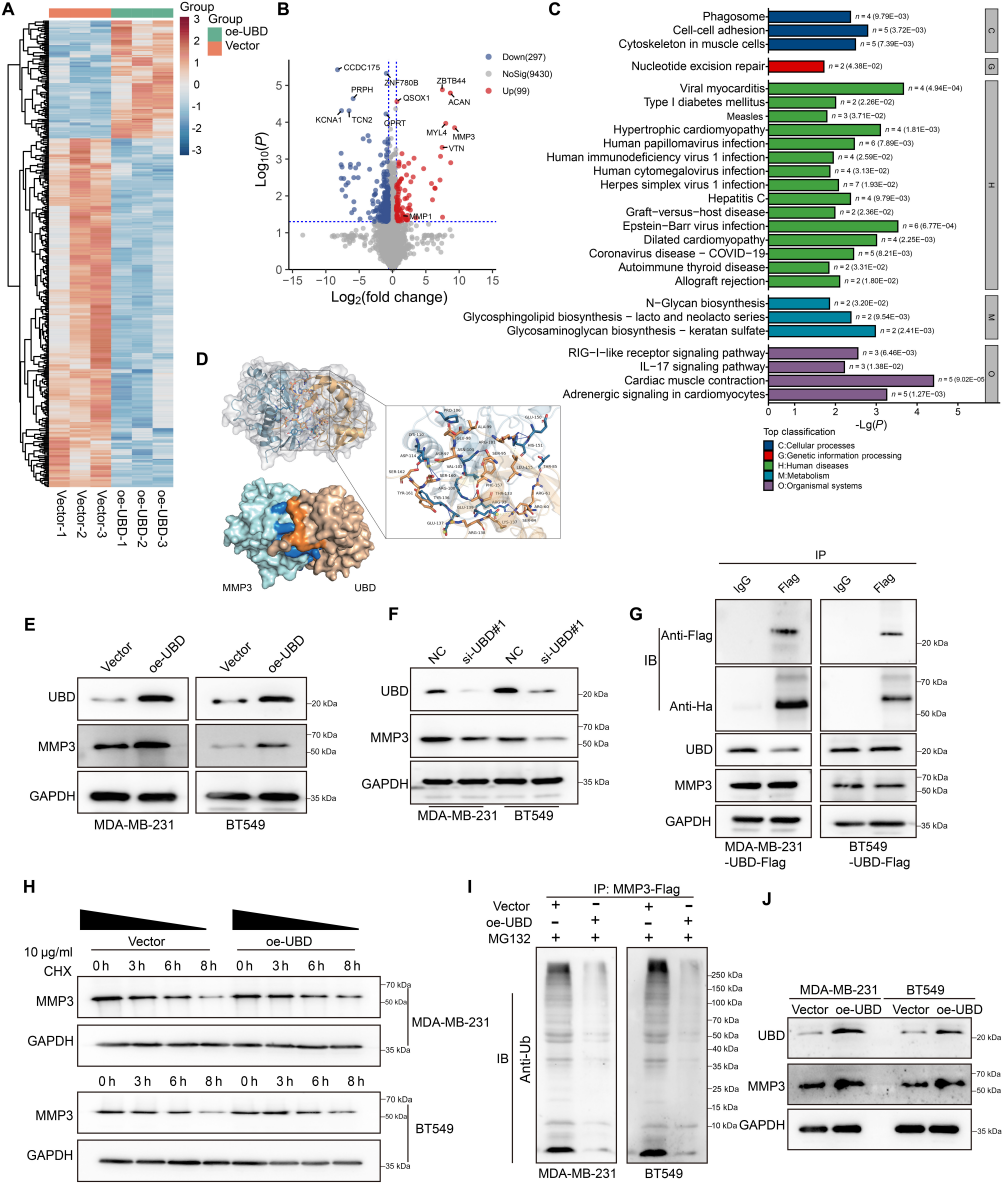
UBD stabilizes MMP3 protein in TNBC cells. (A and B) Differential heatmap and volcano plot analysis were used to identify differentially expressed proteins. (C) KEGG analysis was conducted to determine the enriched signaling pathway differential proteins. (D) PLIP analysis of the HDOCK-predicted UBD–MMP3 complex. Key interactions, including hydrogen bonds, hydrophobic contacts, and salt bridges, are primarily mediated by residues in the C-terminal UBL domain of UBD and the surface residues of MMP3, indicating the potential binding interface. (E and F) Western blot analysis to detect UBD and MMP3 expression levels. (G) Co-IP assay to analyze the interaction between UBD and MMP3. (H) MMP3 protein stability assay analyzing MMP3 expression levels at different time points after CHX treatment. (I) Intracellular ubiquitination of MMP3 was assessed using a ubiquitination assay following UBD manipulation.

We identified UBD downstream regulatory proteins through proteomic analysis in UBD-overexpressing cells. As shown in Fig. 4A and B, differential heatmap and volcano plot analysis revealed significant changes in protein expression upon UBD overexpression. Moreover, Kyoto Encyclopedia of Genes and Genomes (KEGG) pathway analysis of dysregulated proteins in UBD-overexpressing cells indicated significant enrichment in cell–cell adhesion pathways (Fig. 4C). Additionally, proteins interacting with UBD were captured via immunoprecipitation (IP) and subsequently analyzed by mass spectrometry (MS). Through integration of proteomic differential proteins and MS data, 7 overlapping candidate proteins were identified (Fig. S4E). Silver staining revealed a distinct band at approximately 54 kDa (Fig. S4F), and the different peptides were separated from the sample along with their relative abundance (Fig. S4G). Given its molecular weight of around 54 kDa, MMP3 was selected for further validation.

MMP3, also called stromelysin-1, showed marked up-regulation in the UBD-overexpressing group. Notably, as a member of the MMP family, MMP1 is also up-regulated in the UBD high-expression group. To explore the molecular interactions between MMP3 and UBD, we first predicted the structure of UBD and processed it using PyMOL, highlighting the structural domains, including the N-UBL domain, flexible linker, and C-UBL domain, in the schematic model (Fig. S4H). Then, the HDOCK server identified the optimal docking conformation of UBD-MMP3, with a docking score of −217.54, indicating a strong binding affinity and suggesting the formation of a stable complex (Fig. S4I). To further characterize the interaction interface. Protein–ligand interaction profiler (PLIP) analysis was performed on the predicted docking model (Fig. 4D). The results revealed that the interaction is mediated by multiple hydrogen bonds, hydrophobic contacts, and salt bridges, predominantly involving residues from the C-terminal UBL domain of UBD and the key surface residues of MMP3, providing structural insight into the potential binding mechanism. Western blot analysis further confirmed that MMP3 expression increased after UBD overexpression, whereas MMP3 expression decreased following UBD knockdown (Fig. 4E and F). Co-immunoprecipitation (Co-IP) assay showed that UBD coprecipitated with MMP3, indicating a physical interaction between the 2 proteins (Fig. 4G). The results of the protein half-life assay indicated that the breakdown of MMP3 protein was lessened due to UBD overexpression (Fig. 4H). The intracellular ubiquitination assay demonstrated that UBD overexpression markedly reduced the ubiquitination level of MMP3, as evidenced by a decreased smear signal detected by the anti-Ub antibody in Flag immunoprecipitates (Fig. 4I). We examined the expression of UBD and MMP3, and found that UBD overexpression led to increased expression of both UBD and MMP3 (Fig. 4I). We conducted a rescue experiment to confirm that UBD promotes TNBC metastasis through MMP3. MMP3 interference with siRNA effectively inhibited MMP3 protein expression (Fig. S4H), and we selected the most efficient si-3 for further experiments. Cell colony formation assays showed that MMP3 interference suppressed the impact of UBD overexpression on cell proliferation (Fig. S5A and B). Scratch and Transwell assays indicated that MMP3 interference reduced the effect of UBD overexpression on cell migration (Fig. S5C to F). Additionally, the rescue experiment demonstrated that UBD overexpression promotes MMP3 expression, which was restored when UBD and MMP3 interference were combined (Fig. S5G and H). Enzyme-linked immunosorbent assay (ELISA) results showed that UBD overexpression increased both proenzyme and active MMP3 levels, but there was no significant change in the active/proenzyme ratio (Fig. S5I). UBD overexpression promotes tumor growth, while MMP3 knockdown inhibits both tumor growth and metastasis (Fig. S6A). Tumor volume and weight were significantly increased in the UBD overexpression group compared to the control group (Fig. S6B and C). H&E staining and IHC showed higher tumor cell density in the UBD overexpression group compared to the other 2 groups (Fig. S6D), with enhanced cell proliferation, as shown by Ki67 staining (Fig. S6E). MMP3 expression was notably higher in the UBD overexpression group and was reduced in the UBD + si-MMP3 group (Fig. S6F and G). UBD expression was also elevated in the UBD overexpression group (Fig. S6H and I). Furthermore, the UBD overexpression group had a higher number of metastatic lymph nodes (Fig. S6J and K). These results suggest that UBD may promote TNBC metastasis by regulating MMP3 protein stability.

### SPIB transcriptionally regulates UBD expression in TNBC cells

**Fig. 5.**
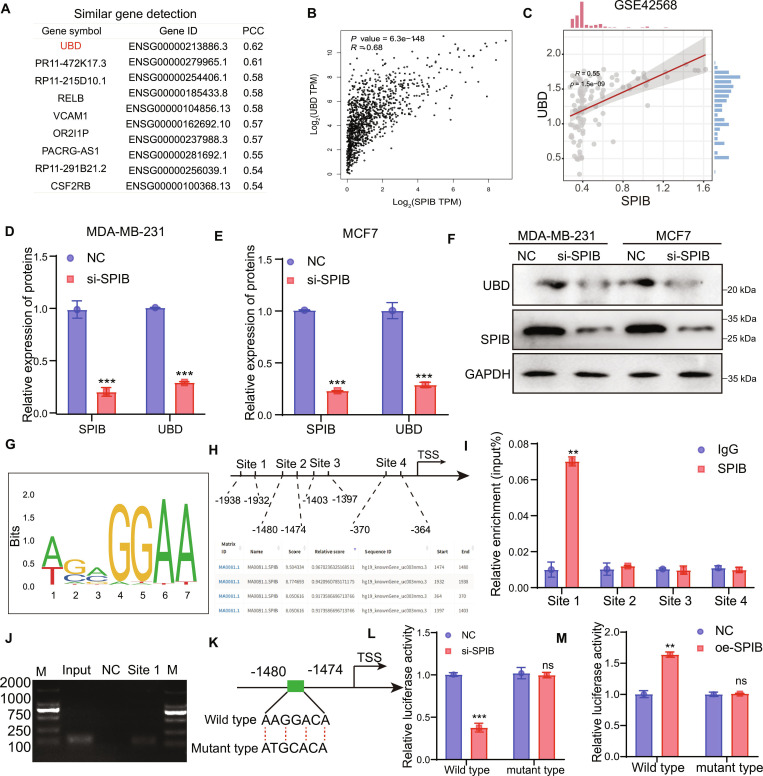
SPIB transcription regulates UBD expression. (A and B) SPIB and UBD have a positive correlation. (C) Correlation analysis between UBD and SPIB expression based on the GSE42568 dataset. (D and E) qPCR was conducted to assess SPIB and UBD expression. (F) WB was used to assess SPIB and UBD protein levels. (G and H) Binding site prediction between SPIB and UBD. (I and J) ChIP assay confirmed SPIB binding to the UBD promoter DNA fragment. (K) Schematic of wild-type and mutant pGL3-basic UBD promoter reporter constructs. (L and M) Relative luciferase activities in MDA-MB-231 cells following si-SPIB or oe-SPIB treatment. Data are shown as mean ± SD. **P < 0.01; ***P < 0.001.

To investigate the upstream regulation of UBD, database analysis indicated a strong positive correlation between UBD and SPIB (Fig. 5A and B). Further analysis using the GEPIA database indicated that SPIB is highly expressed in breast cancer (Fig. S7A), which was further confirmed by the GSE42568 dataset (Fig. S7B). The correlation assessment conducted on the GSE42568 dataset demonstrated a notable positive relationship between the expression levels of UBD and SPIB (Fig. 5C). Additionally, ULCAN database analysis showed that SPIB is abnormally overexpressed in breast cancer, particularly in TNBC tissues (Fig. S7C and D). Furthermore, analysis of the GSE4552 dataset demonstrated that UBD and SPIB are both highly expressed in TNBC, with a statistically significant positive association between their expression levels in tumor tissues (Fig. S7E to G). In addition, we further analyzed the expression of SPIB and UBD using the single-cell RNA sequencing dataset BRCA_EMTAB8107. Our analysis revealed that SPIB is predominantly localized in Mono/Macro and malignant cells of breast cancer, while UBD is primarily found in dendritic cells, Mono/Macro, and malignant breast cancer cells (Fig. S7H and I).

To clarify the biological role of SPIB in TNBC, we designed and synthesized SPIB siRNA. Western blot analysis confirmed that SPIB siRNA effectively reduced SPIB protein expression (Fig. S8A and B). Functional assays indicated that the reduction of SPIB expression markedly decreased the proliferation of TNBC cells, as shown by results from CCK8 and colony formation experiments (Fig. S8C to E). Moreover, results revealed that SPIB depletion reduced cell migration (Fig. S8F to H). SPIB knockdown reduced tumor sphere size and number, indicating impaired self-renewal (Fig. S8I).

Next, qPCR and Western blot revealed UBD expression in SPIB-knockdown TNBC cells. Results indicated that SPIB suppression reduced UBD expression at the RNA and protein levels (Fig. 5D to F). Given SPIB’s function as a transcription factor, we analyzed potential SPIB binding sites within the UBD promoter using UCSC and JASPAR databases. Four candidate binding sites were identified: site 1 (−1,938 to −1,932), site 2 (−1,480 to −1,474), site 3 (−1,403 to −1,397), and site 4 (−370 to −364) (Fig. 5G and H). Chromatin immunoprecipitation (ChIP) assays confirmed that SPIB directly binds to site 1 of the UBD promoter, as indicated by the significantly higher UBD enrichment in the SPIB antibody-precipitated complex compared to the immunoglobulin G (IgG) control. No notable enrichment was detected at sites 2, 3, or 4 (Fig. 5I and J). To further validate this interaction, we constructed a UBD promoter-luciferase reporter plasmid by inserting a 2-kb fragment of the UBD 5′ promoter sequence into the pGL3-basic plasmid, along with a mutant version in which site 1 was disrupted (Fig. 5K). Luciferase activity was markedly diminished in wild-type plasmid-transfected TNBC cells upon SPIB depletion, while cells carrying the mutant construct exhibited no significant difference (Fig. 5L and M).

### SPIB promotes cell proliferation and migration by transcriptionally regulating UBD expression in TNBC cells

**Fig. 6.**
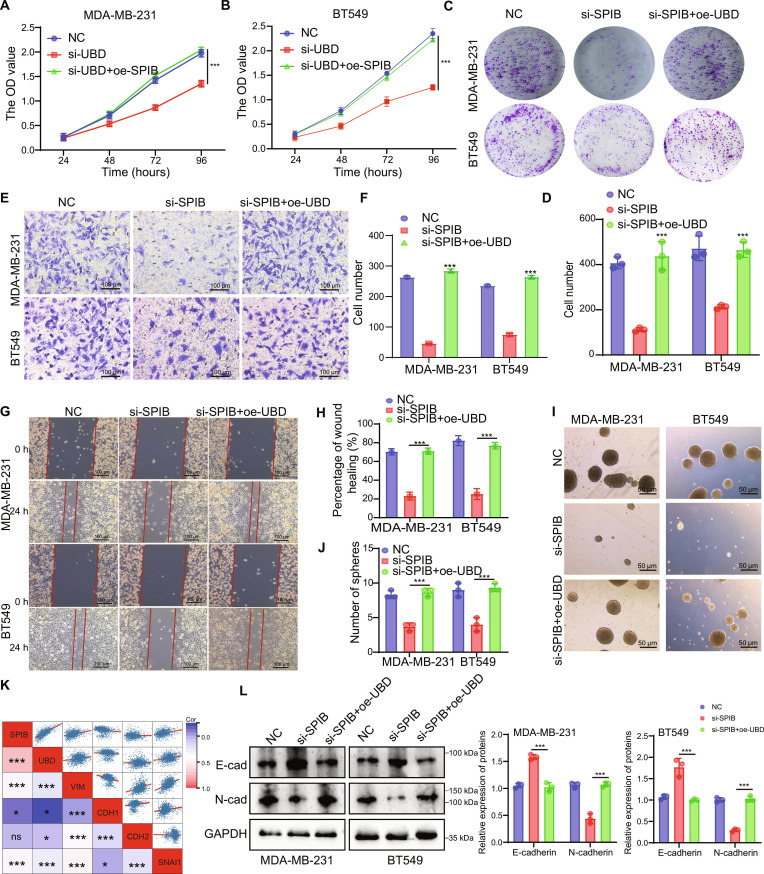
SPIB promotes cell proliferation and migration through UBD. (A to C) CCK8 assay and colony formation assay assessed cell proliferation. (D) Statistical analysis of colony formation results. (E to H) Cell migration was assessed using Transwell and wound healing assays. (I and J) Tumor sphere assay was performed to assess sphere-forming ability. (K) Correlation of UBD, SPIB, and EMT-related protein expression in breast cancer. (L) Western blot assay to detect the expression of EMT-related markers. Data are presented as mean ± SD. *P < 0.05, **P < 0.01, ***P < 0.001.

To assess whether UBD can rescue the effects of SPIB knockdown in TNBC cells, functional assays were performed. Cell proliferation was inhibited following SPIB depletion, as shown by CCK8 and colony formation assays, which was largely reversed by UBD overexpression (Fig. 6A to D). Besides, SPIB knockdown reduced cell migration, which was rescued by UBD overexpression (Fig. 6E and F). Similarly, wound healing assays demonstrated that UBD overexpression restored migration inhibited by SPIB knockdown (Fig. 6G and H). Similarly, tumor sphere formation assays indicated that UBD overexpression restored the reduction in tumor sphere size and number induced by SPIB knockdown (Fig. 6I and J). Further correlation analysis revealed that UBD and SPIB were positively associated with VIM, N-cadherin, and Snail1, but negatively correlated with E-cadherin (Fig. 6K). We further confirmed by WB analysis that SPIB knockdown induces down-regulation of N-cadherin, whereas UBD overexpression effectively reverses its down-regulation. Consistently, SPIB knockdown resulted in the up-regulation of E-cadherin, whereas overexpression of UBD effectively reversed this reduction in E-cadherin expression (Fig. 6L).

### SPIB regulates UBD to promote TNBC metastasis

**Fig. 7.**
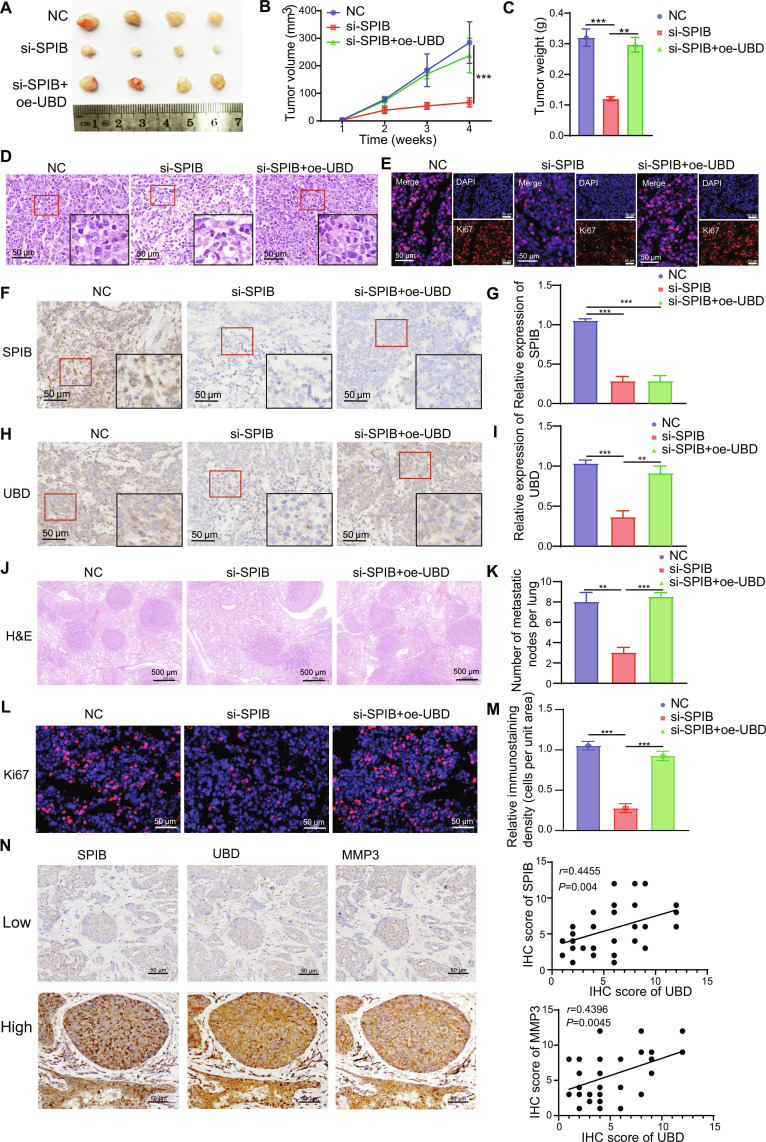
SPIB regulates UBD to promote TNBC metastasis. (A) Representative images of xenograft tumors from different groups (*n* = 4). (B) Tumor growth curves for each group. (C) Analysis of xenograft tumor weights. (D) H&E staining to examine tissue morphology and tumor cell number. (E) Representative Ki67 immunofluorescence images. (F to I) IHC staining for SPIB and UBD across groups. (J and K) H&E staining analysis to quantify metastatic nodules. (L and M) Immunostaining to detect Ki67 expression levels. (N) Representative IHC images illustrating SPIB, UBD, and MMP3 in TNBC tissues with low or high expression (left). Spearman’s correlation analysis was performed (right). Data are presented as mean ± SD. ***P < 0.001.

To further investigate the relationship between SPIB and UBD, we utilized xenograft mouse models to determine whether SPIB promotes TNBC progression via UBD. Tumor size reduction induced by SPIB knockdown was effectively reversed by UBD overexpression (Fig. 7A to C). H&E and immunocytochemistry (ICC) assays confirmed that UBD overexpression restored the proliferative capacity suppressed by SPIB depletion in vivo (Fig. 7D and E). IHC analysis further revealed that while SPIB knockdown significantly reduced UBD expression, UBD overexpression did not affect SPIB levels, suggesting a regulatory hierarchy where SPIB controls UBD expression but not vice versa (Fig. 7F to I).

**Fig. 8.**
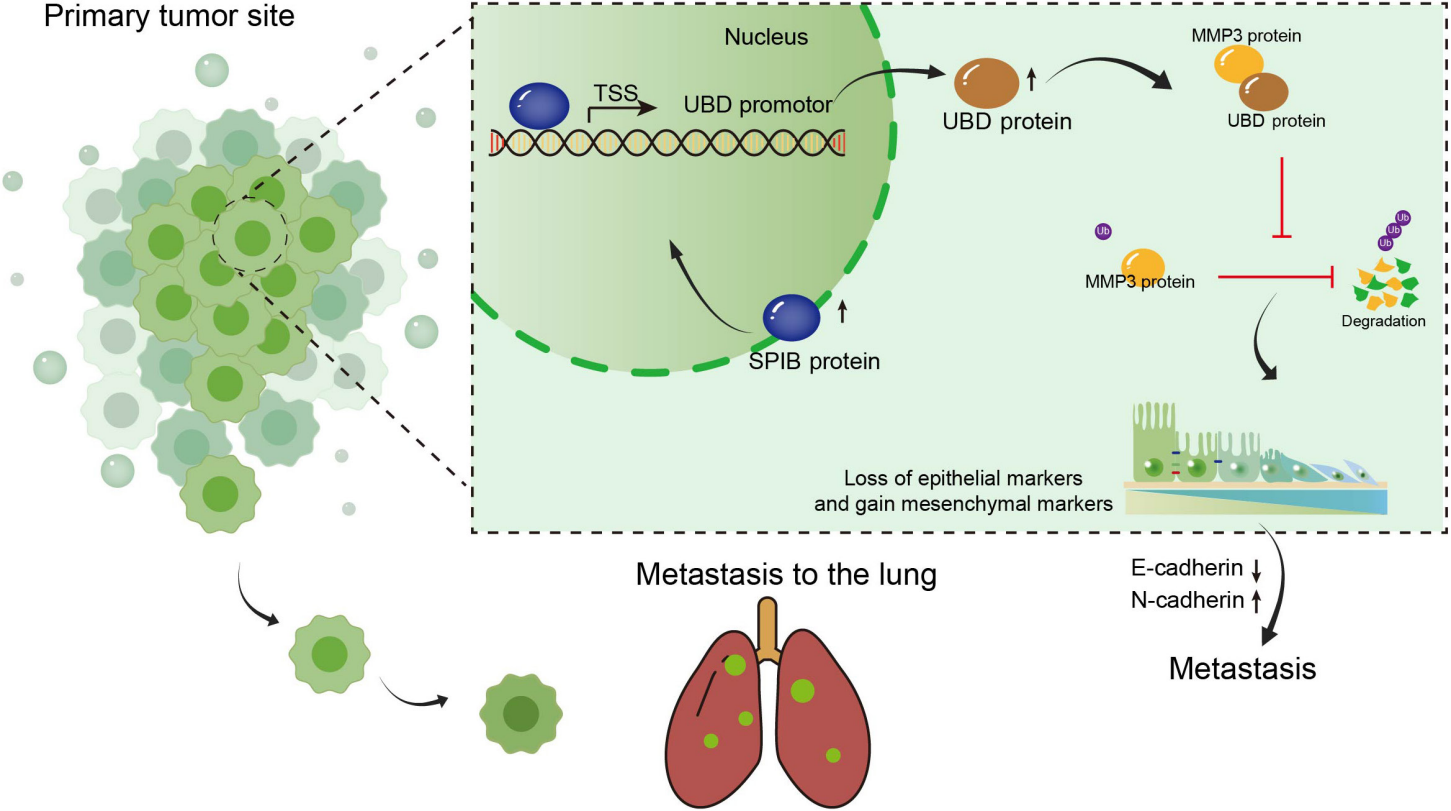
UBD expression was positively correlated with SPIB and MMP3. The diagram explains the biological mechanism of the regulating axis of SPIB/UBD/MMP3 through which lung metastasis of TNBC was induced by promoting the EMT process by up-regulating UBD.

To assess the roles of SPIB and UBD in metastasis, a TNBC lung metastasis model was generated by injecting SPIB siRNA-treated MDA-MB-231 cells alone or combined with UBD overexpression. H&E staining demonstrated that UBD overexpression reversed the suppression of metastatic potential induced by SPIB knockdown (Fig. 7J and K). Additionally, metastatic nodule formation and Ki67 expression were significantly reduced following SPIB knockdown, but these effects were reversed upon UBD overexpression (Fig. 7L and M). Additionally, we observed a positive correlation between the levels of UBD and SPIB (*r* = 0.4455, *P* < 0.01) and MMP3 (*r* = 0.4396, *P* < 0.0045) (Fig. 7N). These results suggest that SPIB up-regulates UBD to promote TNBC proliferation and metastasis (Fig. 8).

## Discussion

TNBC is a clinically aggressive and distinct breast cancer subtype, characterized by the absence or low expression of estrogen receptor (ER), progesterone receptor (PR), and HER2, as determined by IHC [4,5,31]. TNBC makes up roughly 15% to 20% of breast cancer and correlates with unfavorable outcomes, largely because of its tendency for recurrence and metastasis.

Distant metastasis, especially to organs such as the lungs and brain, is the foremost cause of treatment failure and death in TNBC [32]. TNBC metastasizes earlier and more aggressively than other breast cancer subtypes, with limited treatment options after metastasis. While advances in targeted and gene-based therapies have improved outcomes in select patient populations, effective and broadly applicable therapeutic targets for TNBC remain limited [6,8]. UBD is a small ULM in the ubiquitin–proteasome system that contributes to cancer progression [33]. UBD modulates protein stability and influences proliferation, invasion, migration, and immune evasion. It is regularly overexpressed in cancer types including liver, colorectal, and breast cancers [10,34,35]. In the present investigation, we established that UBD expression is considerably enhanced in breast cancer, with a pronounced increase observed specifically in invasive TNBC. Given TNBC’s high metastatic potential—where distant metastases occur in 74% of recurrences, with 36.9% affecting the lungs [36]—the lungs represent the most frequent site of metastatic dissemination among distant organs [37]. IHC analysis confirmed that UBD is abnormally overexpressed in lung metastatic lesions. Functional assays showed that UBD enhances cell proliferation, tumor sphere formation, and migration, whereas its knockdown markedly inhibited tumor growth and reduced lung metastases in vivo. Although tumor sphere assays can provide insights into self-renewal and clonogenic potential, they do not fully capture the heterogeneity and ECM remodeling present in vivo. Future studies employing patient-derived organoid models or other advanced 3D systems will be valuable to further validate the UBD–MMP3 axis in regulating cancer stemness and ECM dynamics. UBD was reported to enhances breast cancer metastasis by stabilizing ZEB2 and facilitating the EMT process [24]. However, the molecular mechanism by which UBD drives TNBC metastasis remains poorly understood.

UBD is a unique ubiquitin-like protein that promotes the proteasomal degradation of substrates in a ubiquitin-independent manner. Notably, previous research demonstrated that UBD can stabilize or degrade substrates in various cancer cells [10]. The attachment of ULMs serves as a critical mechanism regulating protein stability, cellular localization, function, and protein–protein interactions [38]. Disruptions in proteostasis are key contributors to tumor growth and drug resistance [39,40]. In this study, proteomic sequencing and MS analysis identified MMP3 as a downstream target of UBD in TNBC cells. MMP3, also known as stromelysin-1, is an ECM remodeling enzyme involved in metastasis and tumor progression [27,41,42]. MMP3 has been implicated in promoting EMT through various mechanisms, including Rac1b-mediated reactive oxygen species (ROS) production, which enhances malignancy in breast cancer [43,44]. Additionally, MMP3 has been shown to contribute to genomic instability, facilitating neoplastic transformation [25]. Co-IP, silver staining, and in vivo ubiquitination assays demonstrated that UBD binds to MMP3 and inhibits its proteasomal degradation, thereby stabilizing MMP3 protein expression. To further investigate the molecular basis of this interaction, PyMOL was performed to examine the interactions between the receptor and the ligand. These analyses provided detailed information on the interaction forces and key residues involved in the binding domains between the proteins. The results indicate that the C-UBL domain of UBD predominantly participates in the binding with MMP3 and contributes to the stability of the complex structure. UBD has been reported to stabilize multiple proteins, as observed in MAD2, β-catenin, survivin, eEF1A1, and YAP1, suggesting a conserved noncovalent interaction mode that protects substrates from degradation [23,45–47]. Although the precise mechanism by which UBD stabilizes MMP3 remains unclear, insights from recent studies on FAT10ylation suggest a plausible explanation. Ravichandran and Das [48] demonstrated that UBD attachment alters the thermodynamic stability of the substrate in a site-dependent manner. Therefore, it is conceivable that UBD binding may induce local conformational changes in MMP3, potentially masking ubiquitination sites or hindering proteasomal recognition, which could enhance its stability. Further mapping of the UBD–MMP3 interface will help clarify this mechanism.

These results not only demonstrate a physical interaction between UBD and MMP3 but also suggest that UBD may influence the extracellular functions of MMP3. Given these findings, it is reasonable to infer that UBD-mediated stabilization of MMP3 may extend beyond intracellular regulation to influence metastatic colonization. Previous studies have shown that MMP3 plays a key role in lung colonization by degrading ECM components, activating additional proteases, and promoting the establishment of a pre-metastatic niche in the lung [49–51]. Since UBD stabilizes and increases MMP3 expression, the UBD–MMP3 axis may enhance ECM remodeling and create a lung microenvironment that favors tumor cell adhesion and survival. This mechanism may help explain the lung tropism observed in UBD-driven metastasis of TNBC. Our findings suggest that UBD promotes EMT and TNBC metastasis by stabilizing MMP3, highlighting a novel mechanism of TNBC progression.

Beyond posttranslational regulation, we further explored upstream transcriptional mechanisms responsible for the aberrant expression of UBD in TNBC. Further investigation through GEPIA demonstrated that SPIB expression correlates positively with UBD. SPIB, belonging to the E-twenty-six transcription factor family, has been implicated in tumorigenesis [52], immunotherapy response [53], and anoikis resistance [54]. SPIB is up-regulated in several malignancies, including lung cancer [54], B cell lymphoma [55], hepatocellular carcinoma [56], and ovarian cancer [53]. However, it has been reported to exert tumor-suppressive effects in colorectal cancer [57,58]. Furthermore, bioinformatic analyses have indicated that SPIB is involved in tumor immune infiltration [59,60] and is regarded as a prognostic factor in breast cancer [61]. The study offers initial proof that SPIB directly binds to the UBD promoter to influence its transcription in breast cancer, promoting TNBC cell proliferation and migration by increasing UBD levels.

To validate the functional relationship between SPIB and UBD, we performed rescue experiments. Overexpressing UBD counteracted the inhibitory effects of SPIB knockdown on cell proliferation and migration. Besides, in vivo experiments further demonstrated that UBD overexpression counteracted the suppression of tumor growth caused by SPIB knockdown. Using a lung metastasis model, we confirmed that SPIB knockdown reduced lung metastatic burden and Ki67 expression in metastatic nodules, whereas UBD overexpression restored metastasis. Collectively, our findings define a SPIB/UBD/MMP3 signaling cascade that drives TNBC metastasis, providing mechanistic insight into its lung tropism and identifying potential therapeutic targets. From a clinical standpoint, our findings indicate that UBD may function as both a prognostic biomarker and a potential therapeutic target in TNBC. Elevated UBD expression in primary and metastatic tumors is associated with aggressive clinicopathological features, suggesting that its assessment could help identify patients at increased risk of distant relapse. Because UBD stabilizes MMP3 by preventing its ubiquitin-dependent degradation, disrupting UBD itself or its interaction with MMP3 may offer a new approach to limit ECM remodeling and metastasis. Future studies should define the structural basis of the UBD–MMP3 interaction and explore small molecules or peptides capable of interfering with this binding. Validation of UBD and MMP3 as prognostic markers in larger TNBC cohorts will also be important for translating these mechanistic insights into clinical use.

In summary, our study revealed that UBD was up-regulated in TNBC tissues, which was confirmed as oncogenes in TNBC. As a ubiquitin-like molecule, UBD can promote the EMT process and facilitate the metastasis of TNBC by stabilizing MMP3. Its upstream regulation is controlled by the transcription factor SPIB. Moreover, these findings imply that focusing on the SPIB/UBD/MMP3 signaling axis could offer a new strategy for treating TNBC metastasis.

## Materials and Methods

### Sample collection and ethical approval

A total of 80 breast cancer specimens alongside 80 corresponding adjacent normal tissues were procured from the First People’s Hospital of Changde City. All participants consented in writing, and the hospital Ethics Committee approved the study (approval no. 2024-147-01).

### Cell lines, culture conditions, and plasmids

MDA-MB-231and BT-549 were maintained in RPMI 1640 supplemented by 10% fetal bovine serum (FBS) (Inner Mongolia Opcel Biotechnology Co. Ltd.) and incubated under 37 °C humidified conditions with 5% CO₂.

The SPIB, UBD, and MMP3 overexpression plasmids were constructed by Tsingke Technology (Shangsha) Corp. Ltd. The coding sequence (CDS) of SPIB was cloned into the pcDNA3.1 expression vector. The CDS of UBD was cloned into the pcDNA3.1 and pcDNA3.1-3×Flag plasmids to generate the expression constructs oe-UBD and UBD-Flag. The CDS of MMP3 was cloned into the pCAGGS-HA and pcDNA3.1-3×Flag plasmids to generate the expression constructs MMP3-HA and MMP3-Flag, respectively. The pCDNA3.1-HA-Ub plasmid was obtained from Z. Xia’s laboratory at the School of Life Sciences, Central South University. The corresponding primer sequences are in Table S2. Specific siRNAs targeting UBD and SPIB were produced by RiboBio (Guangzhou, China), followed by transfection with Lipofectamine 2000 (11668-027, ThermoFisher, USA).

### Colony formation assay

Colony formation was assessed following established protocols [62]. A total of 1 × 10^3^ cells per dish were grown for 10 d, stained with 1% crystal violet.

### Transwell migration assay

A total of 2.4 × 10^4^ cells in RPMI 1640 were seeded into Matrigel-coated upper chambers (BD Biosciences) for 2 h, while the lower chambers contained 450 μl of RPMI 1640 with 15% FBS. After 8 to 18 h, invaded cells were fixed and stained with 1.5% crystal violet in methanol and enumerated microscopically.

### qRT-PCR

Quantitative reverse transcription PCR (qRT-PCR) was conducted as previously described [58], using glyceraldehyde-3-phosphate dehydrogenase (GAPDH) as the internal control. Primers were obtained from Tsingke Biotechnology (Tsingke, China), with sequences listed in Table S2.

### Western blotting

Western blotting was undertaken using previously reported methods [63]. The following primary antibodies were used: UBD (A5491, ABclonal, China), MMP3 (A22328, ABclonal, China), GAPDH (60004-1-lg, Proteintech, China), E-cadherin (R22490, ZENBIO, China), N-cadherin (R380671, ZENBIO, China), CD44 (R23842, ZENBIO, China), SPIB (220744, ZENBIO, China), HA-tag (AE105, ABclonal, China), DDDDK-tag (ab205606, Abcam, USA), and ubiquitin (A19686, ABclonal, China). The following suitable secondary antibodies were used: goat anti-rabbit IgG (H+L) horseradish peroxidase (HRP) (BS13278) and goat anti-mouse IgG (H+L) HRP (BS12478).

### CSC culture

MDA-MB-231 and BT-549 cells (2,000 cells per well) transfected with siRNA or negative control (NC)-siRNA were plated in low-attachment dishes and grown in Dulbecco’s modified Eagle’s medium (DMEM)/F12 medium (Life Technologies) containing 10 ng/ml epidermal growth factor (EGF), 100 units/ml B27, and 10 ng/ml fibroblast growth factor (FGF). After 14 d, spheres were imaged with a microscope.

### Proteomic analysis, silver staining, and MS

MDA-MB-231 cells were transfected with pcDNA3.1-3×Flag-UBD overexpression or control plasmid. After 72 h, total protein was collected for label-free quantitative proteomic analysis, executed by Shanghai Bioprofile Technology Company Ltd. (Shanghai, China). Flag-tagged UBD was expressed in cells to identify its binding partners. After 48 h, lysates were collected and the supernatant was incubated with Flag-agarose beads overnight at 4 °C. Beads were rinsed 3 times using 1× lysis buffer. After centrifugation and removal of the supernatant, samples were mixed with 2.5× loading buffer and heated to 100 °C for 10 min and then were subjected to sodium dodecyl sulfate–polyacrylamide gel electrophoresis (SDS-PAGE), followed by silver staining (P0017S, Beyotime, China), and liquid chromatography–MS/MS (LC-MS/MS) was conducted using a Q Exactive HF-X mass spectrometer coupled to an Easy nLC1200 system (Thermo Scientific), provided by Shanghai Bioprofile Technology Co. Ltd.

### Co-IP, intracellular MMP3 ubiquitination, and protein half-life assay

Cells were incubated in chilled radioimmunoprecipitation assay (RIPA) buffer with 100× phenylmethylsulfonyl fluoride (PMSF) for 10 min to achieve lysis. The samples were spun at 12,000 rpm for 10 min. The supernatant (30 μg) was used as input, with the leftover sample being incubated with 2 μg of the appropriate antibody and 30 μl of protein A/G-agarose for 24 h at 4 °C. Following 4 washes with 1 ml of cold RIPA buffer, the precipitates were mixed with 2.5× protein loading buffer and boiled at 100 °C for 10 min.

For intracellular MMP3 ubiquitination assay, MMP3-Flag and HA-ubiquitin were cotransfected with either oe-UBD plasmid or empty vector into MDA-MB-231 and BT-549 cells. After 24 h, cells were subjected to a 6-h treatment with 15 μM MG132 (SML1135, Sigma-Aldrich), lysed in NP40 buffer for 15 min, and centrifuged. Once incubated overnight with anti-FLAG beads at 4 °C, the supernatant was washed 3 times, combined with 2.5× loading buffer, and heated at 100 °C for 10 min. An anti-HA antibody facilitated the detection of ubiquitination.

The protein half-life assay for MMP3 was performed as previously described [64]. Briefly, MMP3 and UBD overexpression plasmids were cotransfected into MDA-MB-231 and BT-549 cells. Cells were treated with cycloheximide (CHX) for 0, 3, 6, and 8 h. Total protein was collected at each time point to assess MMP3 levels by Western blot.

### ELISA assay

MDA-MB-231 breast cancer cells were transfected with either a UBD overexpression plasmid or an empty vector as the control. After 48 h of incubation, the culture supernatants were collected for analysis. MMP3 activity in the supernatants was assessed using the MMP3 Activity Assay Kit (Abcam, ab118972), according to the manufacturer’s protocol.

### H&E and IHC

Tumor and lung tissues underwent fixation, paraffin embedding, and sectioning. Sections were prepared for H&E analysis by dewaxing, rehydrating, and staining them with H&E. IHC staining involved overnight incubation with primary antibodies at 4 °C and then treatment with secondary antibodies at room temperature for 30 to 45 min; Ki67 antibody was sourced from MXB Biotechnologies and scored as previously reported [58]. Immunoreactive score (IRS) system was applied, which is widely used for semiquantitative IHC evaluation [65]

### Animal experiments

Subcutaneous transplantation tumor and lung metastasis TNBC models were established using 4-week-old female BALB/C nude mice. All animal experiments received approval from the Animal Care Committee of Central South University (approval no. CSU-2024-0193). For xenografts, 5 × 10^6^ MDA-MB-231 cells transfected with SPIB-siRNA or NC-siRNA (10 nM) were subcutaneously injected thrice weekly for 3 weeks. Tumor volume was estimated as length × width^2^/2, and tumor weight was recorded. In the lung metastasis model, 2 × 10^6^ transfected cells suspended in 100 μl of phosphate-buffered saline (PBS) were administered through the tail vein. After 2 months, mice were sacrificed, and lung tissues were harvested for H&E staining [62].

### ChIP assay

ChIP assays were conducted with the Abcam ChIP Kit (500) following the manufacturer’s instructions. Anti-SPIB (1:1000, #220744, ZENBIO) was used to immunoprecipitate chromatin. Precipitated DNA fragments were analyzed by qPCR and agarose gel electrophoresis (120 V, 30 min). ChIP primers are listed in Table S2.

### Dual-luciferase reporter assay

Sequences from the SPIB and UBD promoter regions, reaching up to 2,000 base pairs before the transcription start site, or their mutant forms, were cloned into the pGL3-basic vector. The Dual-Luciferase Reporter Assay System was employed to determine Firefly and Renilla luciferase activities (Promega, USA), following the manufacturer’s instructions.

### Statistical analysis

Data are expressed as mean ± SD. Statistical analyses were performed using SPSS16.0 and GraphPad Prism 9.5. Student’s *t* test was applied for group comparisons, and gene expression differences in clinical samples were analyzed through a nonparametric rank-sum test. Kaplan–Meier curves analyzed survival outcomes, while the log-rank test determined significance. A *P* value under 0.05 indicated statistical significance.

## Data Availability

The datasets used and analyzed in this study are provided in the article or Supplementary Materials, and additional data can be obtained from the corresponding authors if needed.
