# Supplementary material for: Ubiquitin D Promotes Lung Metastasis by Stabilizing MMP3 in Triple-Negative Breast Cancer
Source: Research (Wash D C). 2026 Jan 23;9:1065. doi: 10.34133/research.1065 (PMC12828796; doi:10.34133/research.1065)
Supplement: Supplementary 1 — Figs. S1 to S8 Tables S1 to S3 [file research.1065.f1.docx]

Supplementary Figure 1


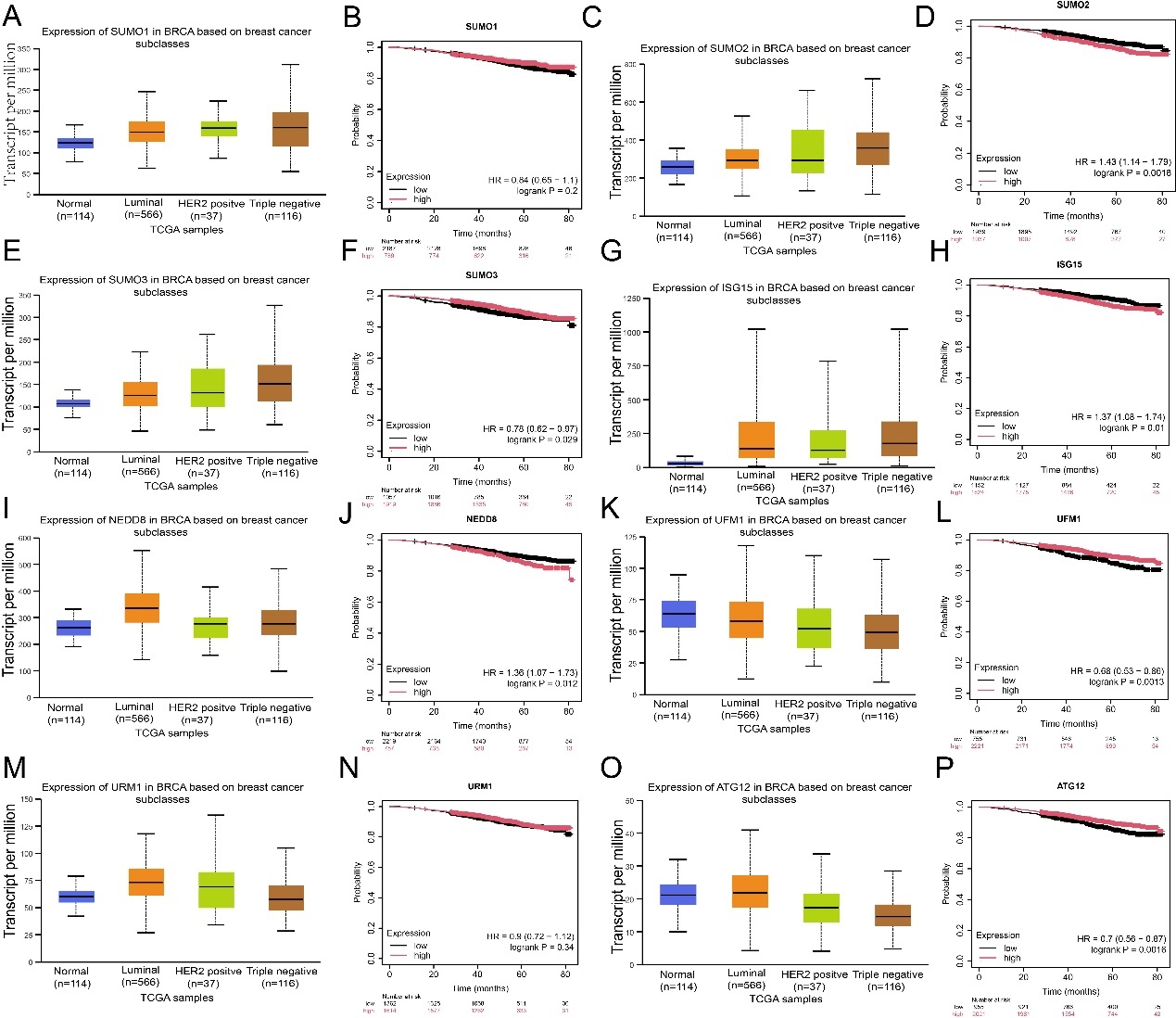


**Supplementary Figure 1**. The UALCAN database and Kaplan-Meier Plotter database was used to analysis the expression and clinical significance of ULMs (SUMO1, SUMO2, SUMO3, ISG15, NEDD8, UFM1, URM1 and ATG12).

Supplementary Figure 2


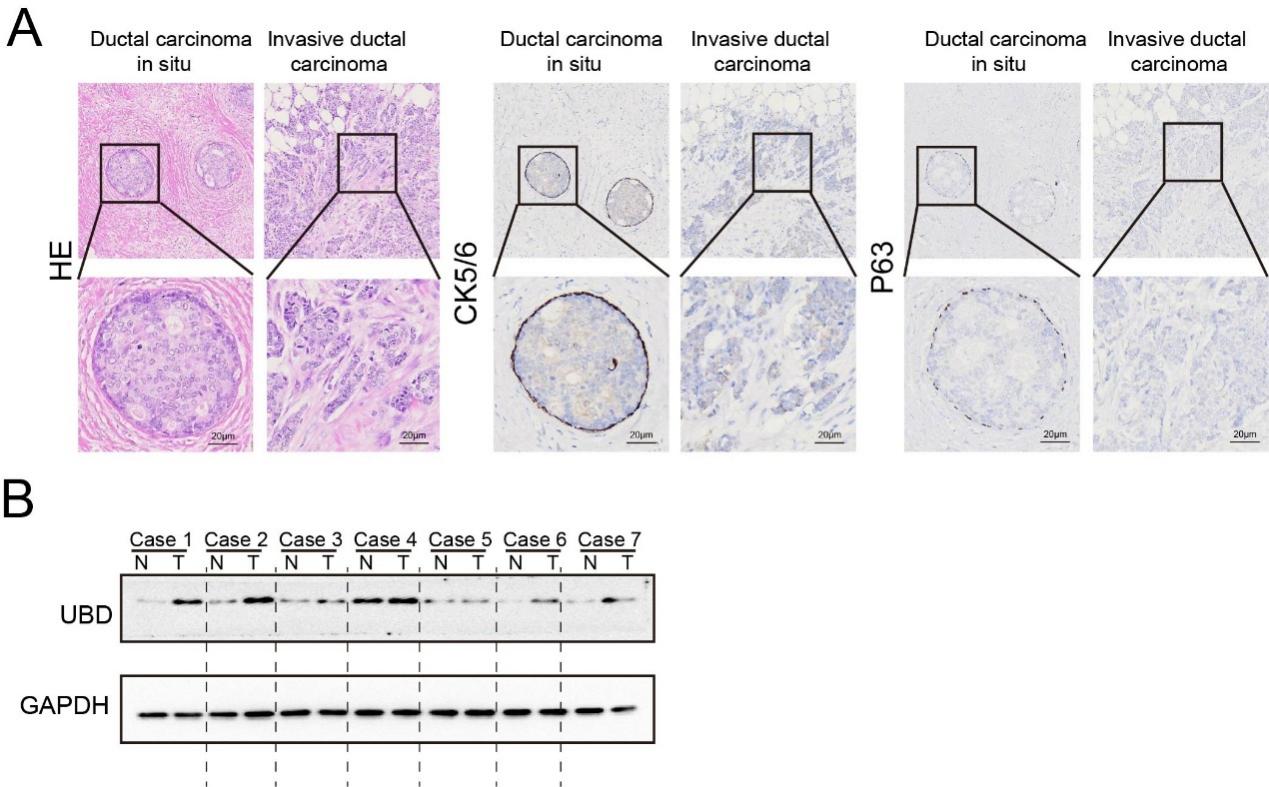


**Supplementary Figure 2**. Identification of different histological subtypes of breast cancer tissues (A) and the protein expression level of UBD in breast cancer tissues (B).

Supplementary Figure 3


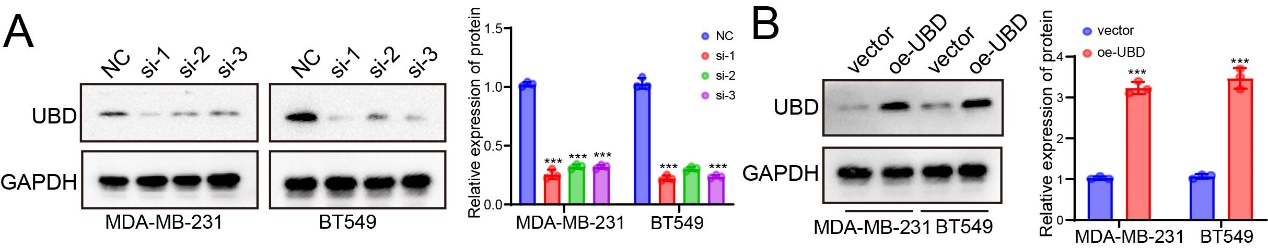


**Supplementary Figure 3**. The silencing efficiency of UBD was evaluated using three specific siRNA sequences.

Supplementary Figure 4


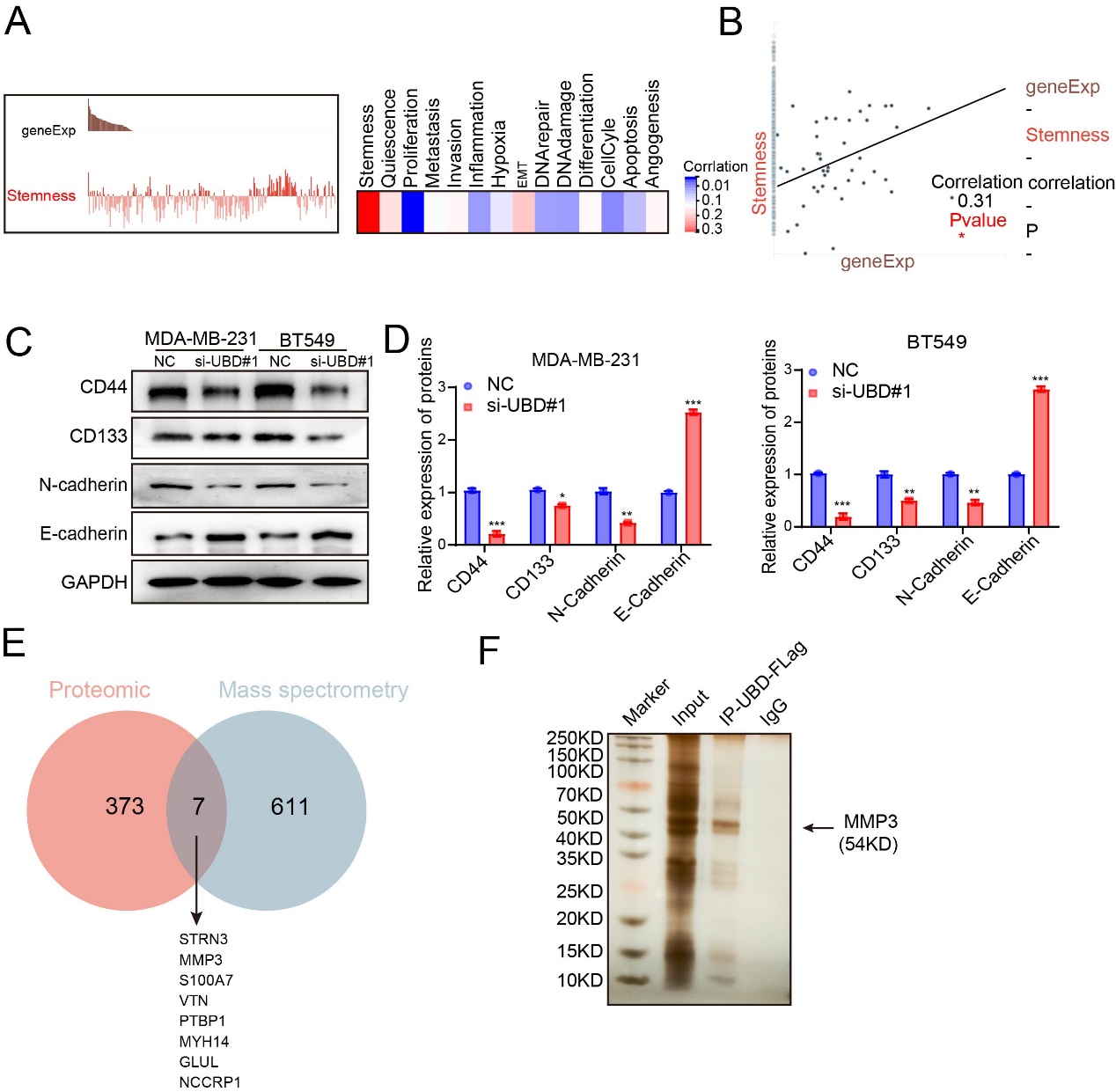


**Supplementary Figure 4**. UBD was associated with tumor stemness. (A-B) CancerSEA database was used to analysis the relatioship between UBD and stemness in breast cancer. (C-D) knockout of UBD effect of tumor stemness and EMT related molecule markers. (E) Venn diagram analysis of overlapping proteins between proteomic and mass spectrometry data. (F) Silver staining analysis of UBD-interacting proteins. *p<0.05, **p<0.01, ***p<0.001.

Supplementary Figure 5


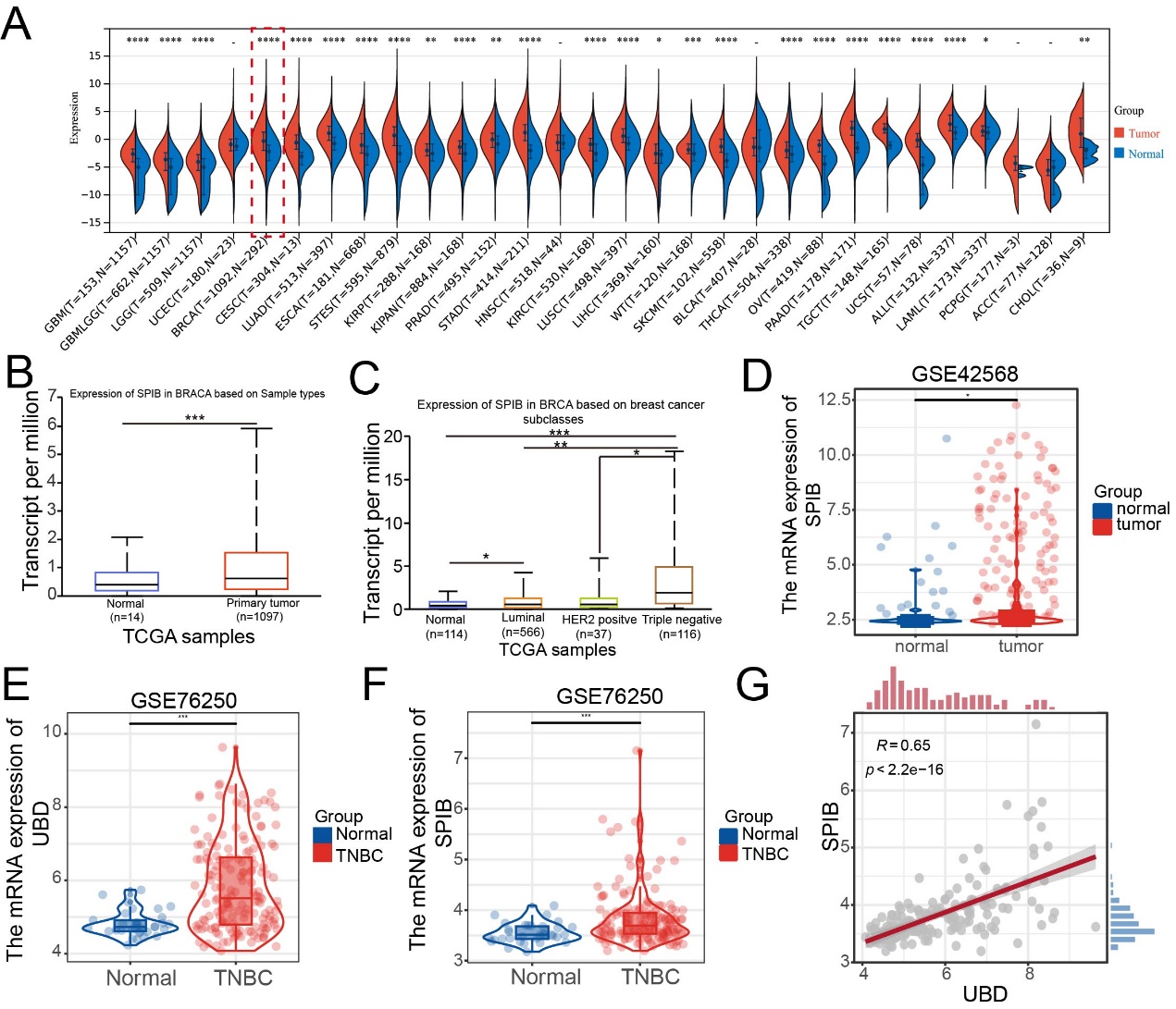


**Supplementary Figure 5**. **The expression levels of UBD and SPIB exhibit a positive correlation in TNBC.** (A)SPIB expression in different cancers; (B) The expression of SPIB in GSE42568 dataset; (C-D) UALCAN database was used to analyze the expression SPIB in subclasses type of breast cancer; (E-F) UBD (E) and SPIB (F) are highly expressed in TNBC. (F) the expression levels of UBD and SPIB show a significant positive correlation in tumor tissues.

Supplementary Figure 6


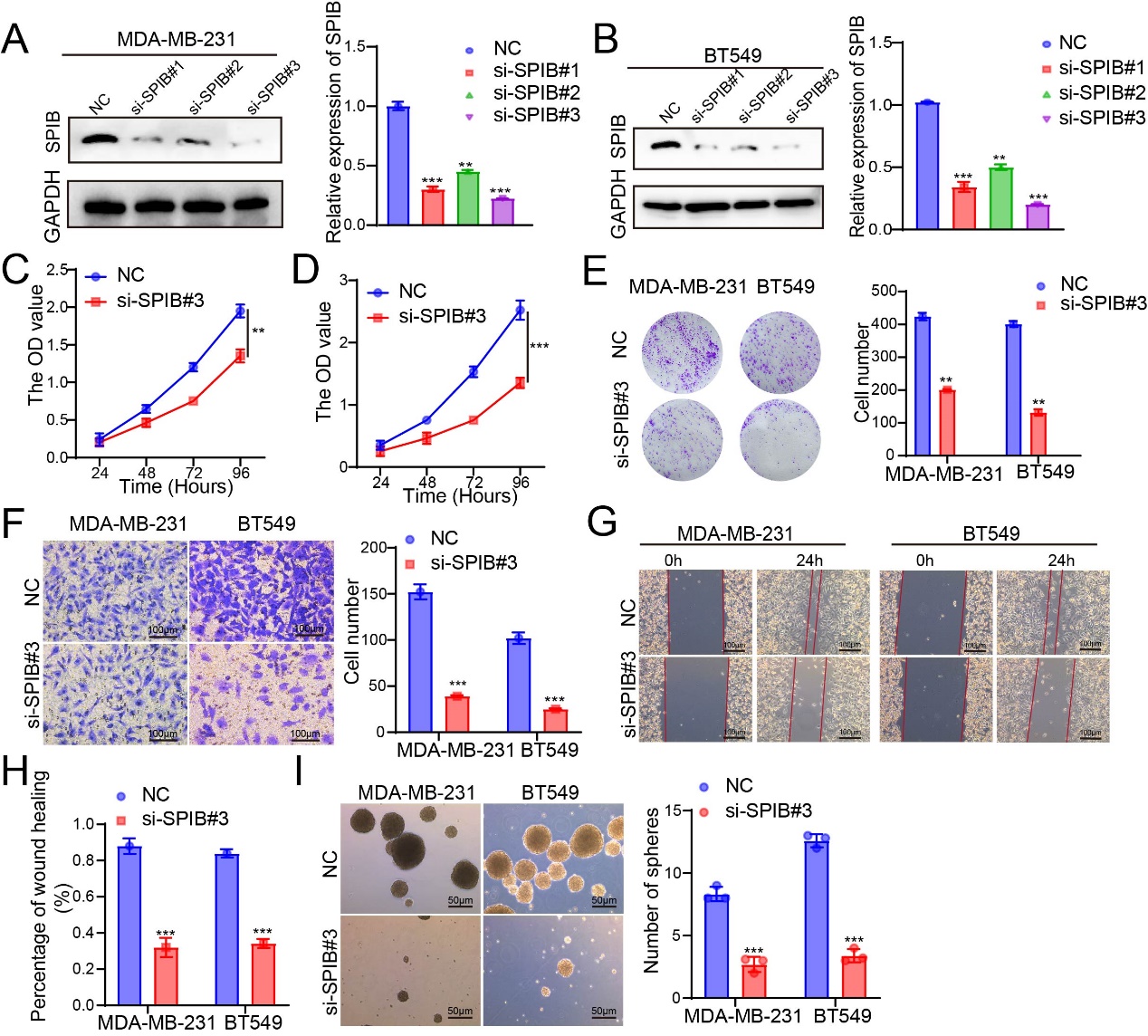


**Supplementary Figure 6**. **Knockdown of SPIB inhibits the proliferation and migration of TNBC.** (A-B) Western blot was used to detect the expression of SPIB in MDA-MB-231 and BT-549; (C-E) CCK8 and Colony formation assay was used to evaluat the effects of SPIB interference on proliferaion. (F) Transwell assay detect the cell invasion in NC and sipSPIB group; (G-H) Wound healing assay was used to analyze the effect of UBD on migrative ability. (I) Tumor sphere assay was used to analyze the effect of SPIB on maintaining tumor stemness. The data present as means ±SD; *p<0.05; **p<0.01; ***p<0.001;

Table S1. Correlation between UBD expression and clinicopathological feature in 80 TNBC patients

| Characteristic |  | All cases | UBD |  | Chi-square | P value |
| --- | --- | --- | --- | --- | --- | --- |
| All cases |  | 80 |  |  |  |  |
|  |  |  | low | high |  |  |
| Age | <50 | 38 | 20 | 18 | 0.001 | 0.580 |
|  | ≥50 | 42 | 22 | 20 |  |  |
| Menopausal | Premenopausal | 35 | 20 | 15 | 1.754 | 0.185 |
|  | Postmenopausal | 45 | 19 | 26 |  |  |
| Grade | II | 37 | 13 | 24 | 2.076 | 0.150 |
|  | III | 43 | 22 | 21 |  |  |
| TNM stage | I | 29 | 20 | 9 | 6.572 | 0.01* |
|  | II/ III | 51 | 15 | 36 |  |  |
| Ki67 | <30 | 30 | 16 | 14 | 5.134 | 0.023* |
|  | >30 | 50 | 14 | 36 |  |  |
| Distant metastasis | Negative | 60 | 31 | 29 | 4.310 | 0.038* |
|  | Positive | 20 | 5 | 15 |  |  |

* Means p<0.05; *** means p<0.001;

Table S2 Primer sequence

| Name | Forward primer (5’-3’) | Reverse primer (5’-3’) |
| --- | --- | --- |
| SPIB | CCCTATGAAGCCTTCGACCCG | GCATATGCCGGGGGAACCA |
| UBD | CTTGTGGAGTCAGGTGATG | CCATTGCAAGTCACAATCTG |
| Site 1 | GAGATCTGTCTCTGAAAGAA | TGTCCCAGACACCTTGG |
| Site 2 | AGACACAGCTAGACAT | TGCAAACCTCATTCCA |
| Site 3 | CAGAAGCAGATGCTGGTGCC | TTTGCTGTTTTAGTC |
| Site 4 | TGCTGAAACAGTAAATA | TGCACAAATGAATTA |
| GAPDH | AGAAGGCTGGGGCTCATTTG | AGGGGCCATCCACAGTCTTC |
| PCDNA3.1-SPIB | AAGCTTATGCTCGCCCTGGAGGCTGCAC | GGATCCTCAGGCCCGGCGGACTGCAGG |
| PCDNA3.1-3×Flag-UBD | AAGCTTGCCACCGACTACAAGGACGACGATGACAAGGGAGGTGGCATGGCTCCCAATGCTTCCTGCC | GGATCCTCACCCTCCAATACAATAA |
| PCDNA3.1 -UBD | AAGCTTATGGCTCCCAATGCTTCCTGCC | GGATCCTCACCCTCCAATACAATAA |
| PCDNA3.1-3×Flag-MMP3 | AAGCTTGCCACCGACTACAAGGACGACGATGACAAGGGAGGTGGCATGAAGAGTCTTCCA | GGATCCTCAACAATTAAGCCAGC |
| PCAGGS-HA-MMP3 | GAATTCATGAAGAGTCTTCCA | CTCGAGTCAACAATTAAGCCAGC |
